# Neural correlates of taste reactivity in autism spectrum disorder

**DOI:** 10.1016/j.nicl.2018.04.008

**Published:** 2018-04-04

**Authors:** Jason A. Avery, John E. Ingeholm, Sophie Wohltjen, Meghan Collins, Cameron D. Riddell, Stephen J. Gotts, Lauren Kenworthy, Gregory L. Wallace, W. Kyle Simmons, Alex Martin

**Affiliations:** a Laboratory of Brain and Cognition, National Institute of Mental Health, Bethesda, MD, United States; b Center for Autism Spectrum Disorders, Children's National Health System, Washington, DC, United States; c Department of Speech, Language, and Hearing Sciences, The George Washington University, Washington, DC, United States; d Laureate Institute for Brain Research, Tulsa, OK, United States; e School of Community Medicine, The University of Tulsa, Tulsa, OK, United States

**Keywords:** Autism, Taste, Food, Insula, Superior temporal sulcus, fMRI

## Abstract

Selective or ‘picky’ eating habits are common among those with autism spectrum disorder (ASD). These behaviors are often related to aberrant sensory experience in individuals with ASD, including heightened reactivity to food taste and texture. However, very little is known about the neural mechanisms that underlie taste reactivity in ASD. In the present study, food-related neural responses were evaluated in 21 young adult and adolescent males diagnosed with ASD without intellectual disability, and 21 typically-developing (TD) controls. Taste reactivity was assessed using the Adolescent/Adult Sensory Profile, a clinical self-report measure. Functional magnetic resonance imaging was used to evaluate hemodynamic responses to sweet (vs. neutral) tastants and food pictures. Subjects also underwent resting-state functional connectivity scans.

The ASD and TD individuals did not differ in their hemodynamic response to gustatory stimuli. However, the ASD subjects, but not the controls, exhibited a positive association between self-reported taste reactivity and the response to sweet tastants within the insular cortex and multiple brain regions associated with gustatory perception and reward. There was a strong interaction between diagnostic group and taste reactivity on tastant response in brain regions associated with ASD pathophysiology, including the bilateral anterior superior temporal sulcus (STS). This interaction of diagnosis and taste reactivity was also observed in the resting state functional connectivity between the anterior STS and dorsal mid-insula (i.e., gustatory cortex).

These results suggest that self-reported heightened taste reactivity in ASD is associated with heightened brain responses to food-related stimuli and atypical functional connectivity of primary gustatory cortex, which may predispose these individuals to maladaptive and unhealthy patterns of selective eating behavior.

**Trial registration:**

(clinicaltrials.gov identifier) NCT01031407. Registered: December 14, 2009.

## Highlights

• We explored behavioral reactivity to taste in autism spectrum disorder (ASD).
• In ASD subjects, taste reactivity was related to the brain's response to tastants.
• Taste reactivity predicted tastant response in gustatory and social brain regions.
• Taste reactivity predicted connectivity between gustatory and social brain regions.

## 1 Introduction

Selective or ‘picky’ eating habits are a common feature of autism spectrum disorder (ASD) (Cermak et al., 2010; Diolordi et al., 2014; Kuschner et al., 2015; Williams et al., 2000). Nearly two-thirds of children with ASD exhibit some form of selective eating, including such traits as food neophobia, food refusal, and insistence on sameness while eating (Cermak et al., 2010; Williams et al., 2000). Compared to the general population, and other atypically developing groups, those with ASD are far more likely to exhibit these maladaptive eating habits (Berkman et al., 2007). While some degree of selective eating is common in young children, most outgrow this behavior at an early age (Birch, 1999). However, in many individuals with ASD, these atypical eating behaviors never fully resolve (Fodstad and Matson, 2008). This high degree of selective eating can lead to the development of poor diets in children with ASD (Sharp et al., 2013), which in turn can lead to inadequate or improper nutrition and poor health outcomes such as a higher risk for obesity (Phillips et al., 2014). Furthermore, these atypical eating behaviors constitute an added burden on families and caregivers, often leading to significant stress at mealtimes (Anderson et al., 2012).

However, despite the prevalence of these behaviors, very little is known about the neural mechanisms that underlie selective eating in ASD. Many of these behaviors are related to other frequently reported symptoms of ASD such as increased sensory reactivity, including heightened reactivity to taste, smell, and oral texture (Cermak et al., 2010; Kral et al., 2015; Kuschner et al., 2015; Williams et al., 2000). Direct studies of taste perception in ASD demonstrate that the identification of specific tastes, such as sucrose, citric acid, or quinine, is impaired in ASD relative to typically-developing (TD) controls (Bennetto et al., 2007; Tavassoli and Baron-Cohen, 2012). In contrast, taste detection thresholds, measured via electrogustometry on the tongue, are comparable between ASD and controls (Bennetto et al., 2007), suggesting a central, rather than peripheral, deficit in taste perception in ASD. Other studies have identified that the perceived sweetness of sucrose, while not different between ASD and control subjects, is negatively related to the severity of ASD social symptoms (Damiano et al., 2014). These equivocal results are likely due to the wide heterogeneity of symptoms present in individuals with ASD. While atypical sensory processing has recently become incorporated as a diagnostic criterion of ASD in the latest edition of the Diagnostic and Statistical Manual of Mental Disorders (DSM-5), this symptom domain is still extremely broad, encompassing multiple sensory modalities, within which individuals may exhibit either hypo- or hyper-reactivity (Hazen et al., 2014). This highlights the need to examine more than simply group differences in studies of sensory perception in ASD, but also to identify significant relationships between behavioral symptoms specific to the sensory modality in question (e.g. taste or smell) and brain activity associated with the perception of that modality.

As such, heightened reactivity in other sensory domains in ASD, such as auditory, visual, or tactile, has been associated with heightened neural activation to those stimuli within the associated primary sensory cortices (Green et al., 2015; Green et al., 2013; Takarae et al., 2014). This suggests that heightened reactivity to taste in ASD would also be associated with heightened taste response within primary gustatory regions of the brain. One recent fMRI study that examined responses to pictures of palatable foods in fasting adolescents identified that ASD subjects exhibited abnormally increased hemodynamic activity to food pictures in the dorsal mid-insular cortex (Cascio et al., 2012). In neuroimaging studies of TD subjects, the dorsal mid-insula has been identified as a region of primary gustatory cortex (Avery et al., 2015; Ogawa et al., 2005; Small, 2010), which also exhibits specific activation to pictures of foods (Simmons et al., 2005; Simmons et al., 2013; van der Laan et al., 2011) relative to other object categories. While these results are suggestive, much still remains unknown about the neural basis of taste reactivity in ASD. Most importantly, no prior neuroimaging study has examined whether the brain's response to tastants is actually altered in ASD, and whether that response is related to individuals' self-reported taste reactivity.

To address this gap in our knowledge of ASD, we used fMRI to assess brain hemodynamic responses to gustatory stimuli in individuals with ASD without intellectual disability. Additionally, atypical patterns of resting functional connectivity are one of the most frequently reported neuroimaging findings in ASD (Cerliani et al., 2015; Cheng et al., 2015; Gotts et al., 2012), and heightened sensory reactivity in ASD has been associated with heightened functional connectivity between limbic and cortical brain regions (Green et al., 2017; Green et al., 2015). As such, resting state fMRI data were also collected from these subjects to determine if atypical hemodynamic responses to gustatory stimuli in ASD subjects were reflected in atypical patterns of resting functional connectivity.

## 2 Methods and materials

### 2.1 Participants

A total of 21 males diagnosed with ASD and 21 TD males, between the ages of 15 and 29, were included in the study. Participants with ASD were recruited from the Washington, DC, metropolitan area and met Diagnostic and Statistical Manual of Mental Disorders – Fifth Edition (DSM-5) diagnostic criteria for ASD. Parents of participants with ASD received the Autism Diagnostic Interview (Lord et al., 1994); and participants with ASD were administered the Autism Diagnostic Observation Schedule (Lord et al., 2000), modules 3 or 4 by a trained, research reliable clinician. Based on these measures, all participants with ASD met the cutoff for the category designated as “broad autism spectrum disorders” according to criteria established by the National Institute of Child Health and Human Development/National Institute on Deafness and Other Communication Disorders Collaborative Programs for Excellence in Autism (Lainhart et al., 2006). In addition, IQ scores were assessed for all participants, and all full-scale IQ scores were ≥80 as measured by the Wechsler Abbreviated Scale of Intelligence –I or –II. Ethics approval for this study was granted by the NIH Combined Neuroscience Institutional Review Board under protocol number 10-M-0027. The institutional review board of the National Institutes of Health approved all procedures, and written informed assent/consent was obtained for all subjects and/or their parent/guardian, when appropriate. Participants were excluded from taking part in the study if they had any history of neurological injury, known genetic or medical disorders that may impact the results of cognitive testing and/or neuroimaging, prenatal drug exposure, severely premature birth or birth trauma, or any exclusion criteria for MRI. TD participants were also excluded if they had any past or present psychiatric conditions (e.g., depression or anxiety disorders), or current usage of psychotropic medications. See Table 1 for participant demographics.

**Table 1:** Participant demographics.

	ASD (*n* = 21)	TD (*n* = 21)	t (ASD – TD)	*p*
Age	21 ± 3	22 ± 3	0.96	0.34
BMI	27 ± 6	23 ± 3	3.11	<0.01
IQ	110.9 ± 13.8	119.52 ± 9.93	−2.32	<0.03
AASP – taste reactivity	11 ± 4	8 ± 3	2.67	<0.01
AASP – sensory sensitivity	32 ± 8	25 ± 8	2.60	<0.01
Sucrose molarity	0.38 ± 0.11	0.39 ± 0.11	0.33	0.74
ASA24 - HEI	46 ± 17	50 ± 11	0.81	0.42
ASA24 – Kcal consumed	2740 ± 840	2378 ± 631	1.51	0.14

AASP – Adolescent/Adult Sensory Profile; ASA-24 – Automated Self-Administered 24-hr dietary recall.

### 2.2 Experimental design

Participants completed fMRI scans during two outpatient visits to the National Institutes of Health Clinical Center in Bethesda, MD. Participant sessions were split into two visits in order to accommodate the time required for multiple behavioral assessments, the taste assessment, and the imaging tasks. On the first day, subjects completed behavioral assessments followed by an anatomical MRI and an 8-minute eyes-open resting-state scan. On the second visit, subjects completed our Food Picture and Gustatory Mapping tasks during scanning.

### 2.3 Behavioral assessments

Prior to imaging, participants completed the Adolescent/Adult Sensory Profile (AASP), a self-report measure used to identify sensory processing patterns that impact everyday functioning (Brown, 2002). To measure taste reactivity specifically, four items from the taste/smell and touch sub-sections of the AASP were used (items 2, 5, 7, & 34, which assess food neophobia and reactivity to strong tastes and food textures). While these items assess different aspects of sensory seeking/responsiveness, previous research in an expanded population of ASD and TD adolescents/young adults indicated that ASD subjects self-reported greater food neophobia and texture sensitivity and lower preference for strong tasting or spicy foods than TD controls (Kuschner et al., 2015). These differences were specific to the modality of taste, as they remained when controlling for global sensory sensitivity. For the present analyses, these items were combined (reverse scored where appropriate) and used as a composite measure of taste reactivity for ASD subjects and controls. In order to measure caloric intake prior to scanning and overall dietary health, participants also completed the Automated Self-Administered 24-Hour Dietary Assessment Tool (ASA-24), a web-based tool used to collect dietary intake information from the past 24 h (Subar et al., 2012).

### 2.4 Imaging tasks

During the Food Picture task (Simmons et al., 2016; Simmons et al., 2013), subjects saw pictures of foods and non-food objects presented against a gray background. To ensure that the subjects attended to all pictures, they were required to press a button whenever two consecutive objects had the same name (exemplar repetition). Subjects performed the Food Picture task during three 317.5 s (5 min 17 s) scanning runs. See Supplemental materials 1.1.2 for additional task details.

During the Gustatory Mapping task (Avery et al., 2017; Avery et al., 2015; Simmons et al., 2013), subjects received tastant solutions, delivered by a pneumatically-controlled MR-compatible tastant delivery system, while undergoing fMRI scanning. Prior to scanning, subjects underwent a taste assessment to determine which concentration of each solution would be used in the scanner (see Supplemental Materials 1.1.1 for details). Subjects received 0.4 mL of a ‘standard’ (0.2M) sucrose solution, a preferred ‘sweet’ sucrose solution (the resultant sweet solution from the pre-scanning taste assessment), and a ‘neutral’ (artificial saliva) solution. The standard sucrose solution was included as a comparison condition in which all subjects received a common sucrose solution irrespective of preference. The average ‘preferred’ sucrose solution was 0.4 M sucrose, and did not differ between ASD and TD subjects (*p* = 0.78). The task involved three types of events: tastant delivery, word-cue, and wash/swallow. During scanning, the subject saw a word-cue for 5 s (“sweet”, “standard”, or “neutral”) indicating the imminent delivery of a specific tastant. The cue word was then replaced with the word “TASTE” for 5 s, during which time the subject received 0.4 mL of either the sweet, standard, or neutral solution. Stimulus periods for both ‘sweet’ and ‘standard’ tastants were combined together for subject-level (i.e. first-level) regression analyses, and are hereafter referred to simply as ‘sweet’ tastants. The neutral solution was also used during ‘wash/swallow’ events, which followed tastant delivery, though the subject was not explicitly told these were the same solutions. Subjects performed the Gustatory Mapping task during three 620 s scanning runs. See Supplemental methods 1.1.3 for additional task details, as well as details on MR-imaging parameters (1.2) and fMRI pre-processing (1.3).

### 2.5 Imaging analyses

#### 2.5.1 Gustatory mapping task analyses

To determine if ASD is associated with an atypical neural response to tastants, we compared the activation to sweet vs. neutral tastants between ASD and TD subjects. We compared these tastant responses first within a priori-defined primary gustatory regions of the brain (i.e. the insular cortex). Within our pre-defined insula masks, we localized specific functional regions responsive to all tastants (both sweet and neutral), using the data from all subjects (FDR corrected *p* < 0.001, see Supplemental methods 1.6.1 for details). Next, we performed a group × region analysis-of-variance (ANOVA) to compare the response to sweet vs. neutral tastants between ASD and TD subjects in each of these insula regions. This approach of a priori ROI selection prior to group analysis allows us to minimize the risk of circular inference (i.e. “double dipping” (Kriegeskorte et al., 2009)). Following this, we performed a separate whole-brain voxel-wise analysis, to identify if any brain regions outside of the insula exhibited differences in tastant responses between ASD and TD groups. (see Supplemental methods 1.6.1 for more details).

Separate analyses of covariance (ANCOVA) were used to determine whether self-reported taste reactivity of ASD and TD subjects was related to the neural response to sweet vs. neutral tastants and whether that relationship differed between ASD and TD subjects, either within the insula regions-of-interest (ROIs), or across the whole brain (with a cluster-size correction of *p* < 0.05, see Supplemental methods 1.6.1 & 1.7 for details).

#### 2.5.2 Food picture task analyses

Following this, we performed an ROI analysis on the data from the Food Picture task to determine whether brain regions that exhibited a group × taste reactivity interaction during the Gustatory Mapping task would also exhibit the same interaction when merely viewing pictures of food.

#### 2.5.3 Resting-state functional connectivity analyses

Finally, we sought to examine whether the group differences observed in the relationships between taste reactivity and tastant response in the Gustatory Mapping task were reflected in group differences of the resting-state functional connectivity of ASD and TD subjects. To accomplish this, we performed a seed-based analysis of resting-state functional connectivity, using data obtained during subjects' 8-minute resting fMRI scans. The five brain regions that exhibited significant interactions of group × taste reactivity during the Gustatory Mapping task (see Imaging results and Fig. 2 below) were used as seeds for the functional connectivity analyses. At the group level, we examined the interaction of group and taste reactivity on the functional connectivity between each of our seed regions and the rest of the brain. As above, resultant statistical maps were whole-brain cluster-size corrected for multiple comparisons at *p* < 0.05. See Supplemental materials 1.6.3–1.7 for more details.

## 3 Results

### 3.1 Behavioral results

ASD and TD participants did not differ in age (*p* = 0.34; Table 1). ASD participants had higher body-mass-indices (BMI; *p* = 0.01) and lower IQ (*p* = 0.03) than TD participants, though the mean IQs for both groups were above average (Table 1). To directly determine if these differences impacted our findings, all analyses reported below were recalculated using both BMI and IQ as co-variates (see Supplemental materials). Because these analyses failed to contribute a significant amount of variance to either of these factors, the results reported below do not take these variables into account. ASD participants scored significantly higher than TD participants on the AASP, both for questions assessing taste reactivity (*p* < 0.01), as well as the sensory sensitivity quadrant score (*p* < 0.01). Analysis of ASA-24 data indicated that groups did not differ in kilo-calories consumed in the 24-hrs preceding scanning (*p* = 0.14), nor did groups differ in terms of their Healthy Eating Index (HEI), an overall measure of dietary health (*p* = 0.42). Across all subjects, there was a negative relationship between taste reactivity and HEI (r(37) = −0.42; *p* < 0.004), indicating that individuals reporting greater taste reactivity also reported less healthy diets. This relationship between taste reactivity and diet was significant in the ASD group on its own (r(18) = −0.42; *p* < 0.04), though groups did not differ in this relationship (*p* = 0.88). Groups did not differ in the molarity of their preferred sweet tastant (*p* = 0.80) during the pre-scan taste assessment or in the tastant they received during scanning (*p* = 0.78).

### 3.2 Imaging results

#### 3.2.1 Gustatory mapping task results

**Fig. 1:**
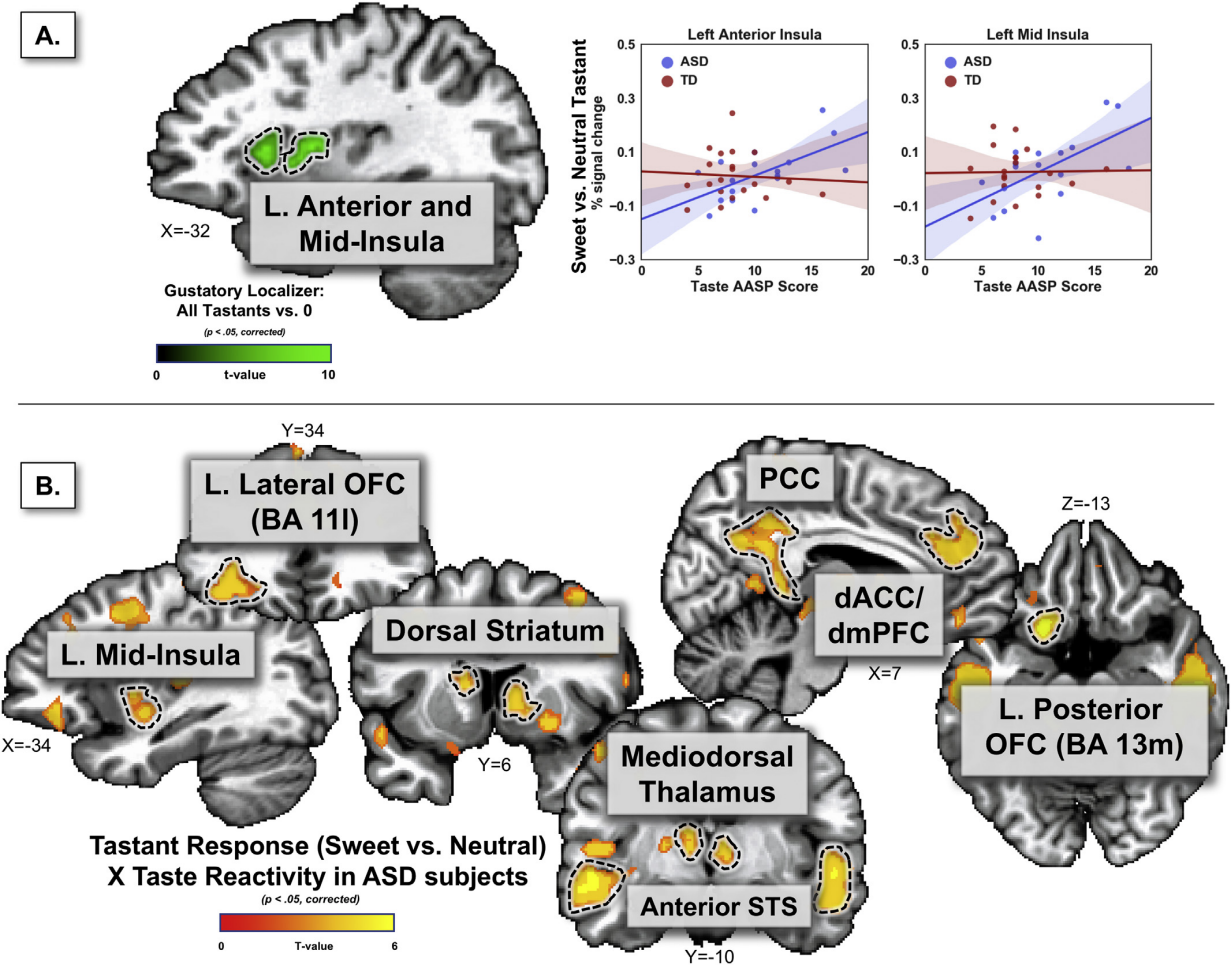
Self-reported taste reactivity is related to the brain's response to sweet tastants in subjects with ASD. A. Within gustatory-responsive regions of left anterior and left mid insula (identified with a gustatory localizer), the response to sweet vs. neutral tastants in ASD subjects (but not TD controls) was positively related to a clinical measure of taste reactivity. B. A whole brain analysis of this relationship identified a host of brain regions associated with gustation, reward, and ASD pathophysiology exhibiting a positive relationship between taste reactivity and tastant response in ASD subjects. No similar relationships were observed in the analysis of TD subjects. AASP – Adolescent/Adult Sensory Profile; ASD - Autism Spectrum Disorder; TD – Typically Developing (control group); OFC – Orbitofrontal Cortex; ACC – Anterior Cingulate Cortex; PCC – Posterior Cingulate Cortex; STS – Superior Temporal Sulcus.

Four distinct taste-responsive clusters were observed in the dorsal anterior and dorsal mid-insular cortex (two per hemisphere) (Fig. 1, Table S1). These regions of the insula, which were responsive to all tastants combined, correspond to those observed in prior neuroimaging studies of taste perception (Avery et al., 2015; Simmons et al., 2013; Small, 2010; Veldhuizen et al., 2011; Yeung et al., 2017).

ASD and TD individuals did not differ in their hemodynamic response to gustatory stimuli within these insula regions (*p* = 0.84) nor was any group × insula region interaction observed (*p* = 0.99). Likewise, no other brain regions exhibited any differences between ASD and TD subjects in the response to sweet vs. neutral tastants.

However, including the data from the self-reported measure of taste reactivity as a covariate (ANCOVA analysis) revealed significant relationships between ASD subjects' taste-related symptoms and tastant response within the insula. Significant effects of taste reactivity were observed within the insula ROIs (*p* < 0.005) as well as significant interactions of group × taste reactivity (*p* < 0.003) and group × insula region × taste reactivity (*p* < 0.001). These interaction effects were due to a positive relationship between taste reactivity and the response to sweet vs. neutral tastants in both regions of the left insula (anterior, r(15) = 0.63; *p* < 0.001; mid-dorsal, r(15) = 0.59; *p* < 0.003) for the ASD, but not TD subjects (Fig. 1). The insula regions in the right hemisphere showed the same interaction pattern but failed to reach significance (anterior, r(15) = 0.34; *p* = 0.20; mid-dorsal, r(15) = 0.40; *p* = 0.08). In a supplemental analysis, these results were replicated using an independent set of insula ROIs from a meta-analysis of human gustatory neuroimaging studies (Veldhuizen et al., 2011), confirming that these results are not due to any bias in ROI selection (see Supplemental materials).

The whole brain analysis revealed multiple brain regions that exhibited a significant positive relationship between taste reactivity and tastant response (sweet vs. neutral) in the ASD subjects (Fig. 1b, Table 2). These included limbic and paralimbic cortical regions such as the left mid-insula, left posterior insula, left lateral orbitofrontal cortex (OFC; BA11l), left posterior OFC (BA13m), and the right ventromedial prefrontal cortex (BA11m). This relationship was also observed in sub-cortical regions such as the striatum – including bilateral dorsal caudate and right ventral putamen, and the bilateral mediodorsal thalamus. Multiple other cortical regions such as the bilateral middle frontal gyrus, bilateral anterior superior temporal sulcus (aSTS), and the dorsal anterior and posterior cingulate cortex also exhibited a positive relationship between taste reactivity and response to sweet vs. neutral tastants in ASD subjects (Table 2). In contrast, within the TD group, the relationship between neural correlates of tastant response and self-rated taste reactivity did not survive statistical thresholding and multiple comparison correction.

**Table 2:** Brain regions exhibiting a positive relationship between taste reactivity and the response to sweet vs. neutral tastants in ASD subjects.

Location (multiple anatomical regions within a cluster are separated by semi-colons)	Peak coordinates			Peak T	Cluster *p*-value	Volume (mm^3^)
	X	Y	Z			
L anterior STS; L dorsal striatum; L ventral putamen; L mediodorsal thalamus; L mid-insula	−51	−19	−2	5.85	<0.01	14,472
L dorsomedial PFC; L dorsal anterior cingulate; L dorsolateral PFC	−3	− + 37	+40	5.32	<0.01	13,888
R anterior STS	+49	−17	−14	6.49	<0.01	11,376
L posterior cingulate	−9	−39	+26	5.95	<0.01	9192
L superior parietal lobe	−7	−31	+44	5.83	<0.01	3520
L posterior OFC (BA 13 m); L lateral OFC (BA11l)	−17	+11	−14	6.24	<0.01	3376
L middle frontal gyrus	−33	+5	+40	4.74	<0.01	3112
L postcentral gyrus	−23	−33	+44	4.31	<0.01	2504
L superior frontal gyrus	−19	+37	+30	4.44	<0.01	2480
R ventral putamen; R dorsal striatum	+23	+11	−2	4.81	<0.01	2232
R ventroposteromedial thalamus; R mediodorsal thalamus	+15	−17	+8	4.52	<0.01	1520
L posterior STS	−47	−55	+16	4.34	<0.01	1416
R middle frontal gyrus	+35	+3	+52	4.48	<0.01	1320
L posterior insula	−47	−55	+16	4.61	<0.01	1128
R postcentral gyrus	+35	+3	+52	3.99	<0.02	728
L paracentral lobule	−47	−9	+6	3.89	<0.03	704
L cuneus	−15	−89	+10	5.53	<0.03	680
L parahippocampal gyrus	−15	−27	−14	5.10	<0.04	608
L ventromedial PFC (BA 11 m)	+11	+39	−8	4.11	<0.04	584
R postcentral gyrus	+49	−27	+48	4.20	<0.04	576

L – left; R – right; PFC – prefrontal cortex; OFC – orbitofrontal cortex; STS – superior temporal sulcus.

**Fig. 2:**
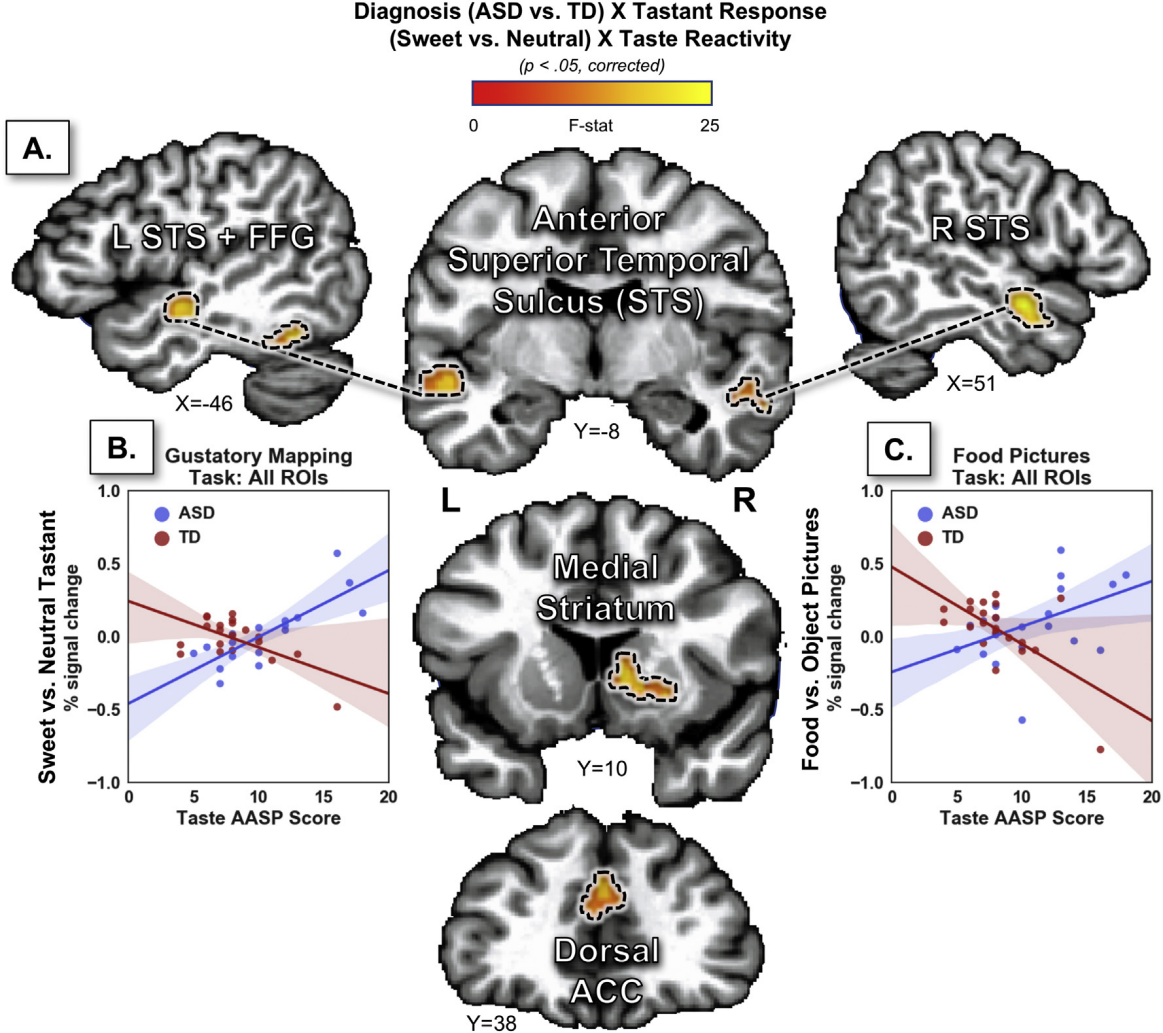
Brain regions exhibiting group differences in the relationship between taste reactivity and the response to food stimuli. **A**. Several brain regions (coronal and sagittal slices below) were identified in a whole-brain analysis examining the interaction of group (ASD vs. TD) by taste reactivity on the response to sweet vs. neutral tastants. **B**. (Left scatterplot) The beta weights, extracted and averaged across each of those brain regions, demonstrates the crossover interaction of tastant response by taste reactivity in ASD and TD groups. **C**. (Right scatterplot) A similar Region-Of-Interest (ROI) analysis using data from the Food Picture task demonstrates that these brain regions exhibit the same crossover interaction effect for the response to food vs. object pictures. AASP – Adolescent/Adult Sensory Profile; ASD - Autism Spectrum Disorder; TD – Typically Developing (control group).

Another subsequent whole brain analysis, which contrasted this brain-behavior relationship between ASD and TD subjects identified five regions exhibiting a significant interaction of group × taste reactivity (Fig. 2, Table 3). These regions included the bilateral aSTS, a region of the right medial striatum including the dorsal caudate and the ventral putamen, the left posterior fusiform gyrus, and the dorsal anterior cingulate cortex.

**Table 3:** Brain regions exhibiting an interaction between group and taste reactivity in the response to sweet vs. neutral tastants.

Location	Peak coordinates			Peak F	Cluster p-value	Volume (mm^3^)
	X	Y	Z			
L anterior superior temporal sulcus	−53	−13	−6	22.51	<0.01	1024
R anterior superior temporal sulcus	+51	−3	−12	24.98	<0.01	824
R medial striatum	+9	+11	+4	20.85	<0.05	496
L fusiform gyrus	−39	−47	−20	16.61	<0.05	472
Dorsomedial anterior cingulate; dorsomedial prefrontal cortex	+9	+33	+22	17.34	<0.06	464

L – left; R – right.

#### 3.2.2 Food picture task results

Importantly, an ROI analysis performed on data from the Food Pictures task indicated that these same brain regions also exhibited a significant interaction of group × taste reactivity in the response to food vs. object pictures (*p* < 0.002; Fig. 2c). Individual post-hoc analyses confirmed this effect within each of these five brain regions (Table S2). As in the Gustatory Mapping task data, within these regions, ASD subjects exhibited a positive relationship between taste reactivity and the hemodynamic response to food vs. object pictures.

#### 3.2.3 Resting-state functional connectivity results

**Fig. 3:**
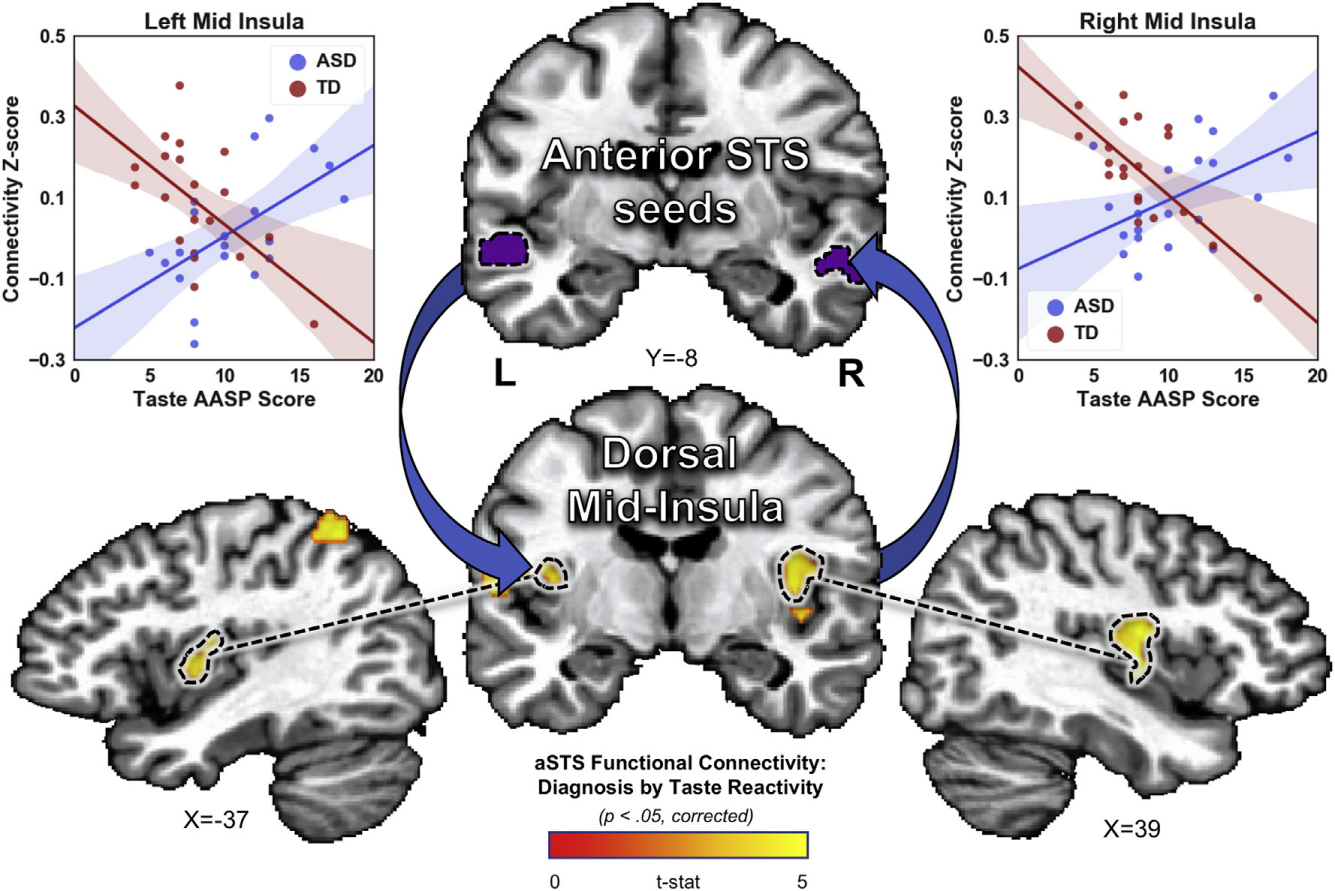
Interactions of diagnosis and taste reactivity on the resting-state connectivity of the anterior superior temporal sulcus. We examined the seed-based connectivity of each of brain region that displayed a significant group by taste reactivity interaction of the response to food stimuli (see Fig. 2a). The bilateral anterior superior temporal sulcus (STS) exhibited a similar interaction in its resting-state connectivity to the bilateral dorsal mid-insular cortex. Scatterplots, included for display purposes only, show the direction of connectivity × taste reactivity relationships within 5 mm spheres around peak voxels in left and right mid-insula. AASP – Adolescent/Adult Sensory Profile; ASD - Autism Spectrum Disorder; TD – Typically Developing (control group).

Finally, analysis of the resting-state connectivity of these five ROIs revealed a significant interaction of group and taste reactivity on the functional connectivity between the bilateral STS and multiple regions of the brain, including regions of the right post-central gyrus, left parietal cortex, and the bilateral dorsal mid-insular cortex (Fig. 3, Table S3).

## 4 Discussion

Selective or picky eating behaviors are highly prevalent in ASD (Williams et al., 2000) and are associated with abnormal reactivity to taste, smell, and oral texture (Cermak et al., 2010; Kuschner et al., 2015). The goal of the present study was to examine the neural correlates of taste reactivity in ASD, including whether individuals with ASD differ from controls in the neural response to tastants.

Adolescents and young adults with ASD, relative to neurotypical controls, reported significantly greater reactivity to taste, as measured by taste-specific items from the Adolescent/Adult Sensory Profile. This finding was previously observed in an expanded population of subjects with ASD (Kuschner et al., 2015). In group comparisons, we observed no differences between ASD and TD subjects in the neural response to sweet (vs. neutral) tastants. However, unlike control subjects, taste reactivity in ASD subjects was positively related to their response to sweet (vs. neutral) tastants in primary gustatory cortex. For the ASD subjects, the more taste-related symptoms they reported, the greater their tastant response in left anterior and dorsal mid-insular cortex. This finding is in accord with previous evidence in ASD that heightened reactivity to other sensory modalities, such as sight, sound, and touch, is accompanied by stronger activation to those modalities in primary sensory cortices (Green et al., 2015; Green et al., 2013).

We also observed this positive relationship between taste reactivity and sweet tastant response in other brain regions previously associated with gustatory stimulation (Veldhuizen et al., 2011; Yeung et al., 2017), such as the left orbitofrontal cortex - including anterior lateral and posterior medial sub-regions also involved in separate aspects of food-value based decision making (Murray et al., 2015; Rudebeck and Murray, 2014); bilateral regions of the dorsal caudate also associated with increased dopamine binding following sucrose ingestion (Small et al., 2003), and a region of the right mediodorsal thalamus. This pattern of activations suggests that, in addition to heightened sensory activation to tastants, heightened taste reactivity in ASD may also result in heightened affective and hedonic responses to consumption of palatable foods.

The clinical relevance of these results is underscored by the finding that, across all subjects, greater taste reactivity was related to a poorer quality diet, as measured by the Healthy Eating Index of the ASA-24. Lower scores on the HEI accompany higher degrees of obesity (Guo et al., 2004) and lower cardio-metabolic health (e.g. elevated blood-pressure or cholesterol level) (Camhi et al., 2015). Thus, heightened taste reactivity, accompanied by heightened sensory and affective responses to taste, as well as unhealthy dietary patterns, may predispose individuals with ASD to a host of adverse health outcomes such as type-2 diabetes and cardiovascular disease (Schwingshackl and Hoffmann, 2015). This suggests that children with ASD who exhibit high levels of taste reactivity might benefit from nutritional counseling as a supplement to traditional therapeutic strategies.

Interestingly, we also observed this relationship between tastant response and taste reactivity within a wider network of brain regions including the bilateral anterior superior temporal sulcus (STS), the dorsal anterior cingulate cortex, and the fusiform gyrus. This network of regions is typically associated with aspects of social cognition (Adolphs, 2001) and multisensory integration (Beauchamp et al., 2004; Beauchamp et al., 2008). These regions have also been implicated in heightened reactivity to auditory, visual, and tactile stimuli in ASD (Green et al., 2015; Green et al., 2013), as well as in the pathophysiology of ASD more generally (Amaral et al., 2008; Bigler et al., 2007; Boddaert et al., 2004; Verhoeven et al., 2010). In the present study, the relationship between taste reactivity and sweet tastant response in these regions differed significantly between ASD and TD subjects, as groups exhibited divergent tastant responses within these regions with increasing taste reactivity. Importantly, these results extend beyond the response to sweet tastants alone, as the same relationships were present in these regions in a separate task using pictures of foods rather than tastants. This suggests that heightened taste reactivity in ASD affects both the direct experience of taste, as well as the learned taste properties associated with a variety of visually-presented foods.

Furthermore, we identified that this interaction of group and taste reactivity was also manifested in the resting-state functional connectivity between the bilateral anterior STS and bilateral regions of primary gustatory cortex in the dorsal mid-insula. The anterior STS harbors reciprocal neural connections to multiple limbic, paralimbic, and sensory association areas of the brain (Barnes and Pandya, 1992; Seltzer and Pandya, 1994), including the insular cortex (Mesulam and Mufson, 1982; Mufson and Mesulam, 1982). The activity of this region of the anterior STS has been associated with multiple processes relevant to social function such as face perception, biological motion perception, and higher-order aspects of social cognition (Adolphs, 2001; Allison et al., 2000; Grossman et al., 2010; Hein and Knight, 2008), all of which are frequently impaired in individuals with ASD (Adolphs et al., 2001; Zilbovicius et al., 2013). In the present study, a greater degree of taste reactivity in ASD was predicted by greater food-related activity of the aSTS and greater functional connectivity between the aSTS and gustatory regions of the dorsal mid-insular cortex. This suggests that heightened reactivity to taste in ASD might result from an overrepresentation of gustatory input within the anterior STS, due to increased (and likely bi-directional) connectivity between that region and primary gustatory regions of the insula. It is noteworthy that significant disturbances in global connectivity involving the anterior STS have been reported in multiple neuroimaging studies of ASD (Cheng et al., 2015; Gotts et al., 2012; Ramot et al., 2017). One possible explanation for these various results would be a widespread functional reorganization in ASD, in which one or more brain regions typically involved in processing social stimuli are re-purposed for the processing of basic sensory (i.e. taste-related) stimuli. This would suggest that disordered connectivity of the aSTS may lead to a diverse set of functional abnormalities, including deficits in social function as well as heightened sensory reactivity that is present in many individuals with ASD.

### 4.1 Strengths and limitations

Strengths of this study include the evaluation of taste reactivity using multiple methods: the Adolescent/Adult Sensory Profile, which provided a clinical self-report (as opposed to parent or caregiver-reported) measure of taste-related behaviors; the pre-scan taste assessment, which provided a measurement of the subjective perception of taste qualities; and the gustatory mapping task, which measured the direct neural response to taste. The specific AASP items used to assess taste reactivity in this study have been shown to reliably discriminate between ASD and TD controls (Kuschner et al., 2015). Within the present study, the composite measure of taste reactivity was significantly related to overall levels of sensory sensitivity and sensory avoidance in ASD and TD subjects, as measured by the AASP. While the AASP is a widely used measure of sensory symptoms in ASD (Dunn and Westman, 1997), it may be better suited to assessing affective responses and compensatory behaviors that occur as a result of abnormal sensory function, rather than sensory function per se (Tavassoli et al., 2014). Though measuring these affective compensatory behaviors may actually have more direct bearing on the study of selective eating in ASD, future studies using measures such as the Sensory Perception Quotient (Tavassoli et al., 2014) may allow a more objective assessment of taste perception in ASD.

Additionally, ASD is typically associated with abnormalities in processing bitter and sour tastes (Bennetto et al., 2007; Tavassoli and Baron-Cohen, 2012), as well as dislike of certain food textures (Kuschner et al., 2015). As this study examined sweet taste specifically, we may be limited in our ability to generalize our results to taste stimuli that individuals with ASD find more aversive. We chose to use sucrose in our gustatory mapping task for mainly practical experimental concerns related to head motion and participant retention. However, we also observed that those same brain regions that exhibited a significant interaction between group and taste reactivity on the taste of sucrose also exhibited the same interaction in the response to food vs. object pictures. As the food pictures used for this study included a variety of foods ranging from highly palatable and sweet foods (e.g. hamburgers, chocolate) to less palatable and healthy foods (e.g. vegetables), this suggests that the present results would generalize beyond simply the response to sweet-tasting foods. Regardless, it will be important for future imaging studies to examine taste reactivity in ASD using multiple different types of tastants, especially in combination with olfactory delivery, as simultaneous multimodal stimuli seem to be particularly aversive for individuals with ASD (Green et al., 2015; Green et al., 2013).

Within the present study, ASD and TD subjects were matched on age and gender. The groups did differ in IQ and body-mass-index (BMI), however. Importantly though, the mean IQ scores for both groups were above average and the tasks were not cognitively demanding. Importantly, neither IQ nor BMI was related to taste reactivity though. We also performed supplemental analyses using both IQ and BMI as covariates (see Supplemental materials) and identified that they had no effect upon our results. However, future studies using participant samples matched on BMI may help to control for metabolic or hedonic effects upon gustatory and food-related brain responses.

### 4.2 Conclusion

The present study examines the neural response to taste stimuli and its relationship to taste reactivity in ASD. Our findings demonstrated that a self-reported measure of taste reactivity in ASD was positively associated with neural responses to sweet tastants as well as food images. In gustatory regions of the insular cortex, as well as brain regions associated with affective and reward processing, the neural response to tastants was positively related to taste reactivity in participants with ASD, but not TD controls. Significant group differences in the relationship between taste reactivity and the neural response to gustatory stimuli occurred in a network of brain regions previously implicated in the pathophysiology of ASD, including the anterior STS and medial prefrontal cortex. These regions exhibited the same pattern of group differences in the relationship between taste reactivity and the response to food pictures. These interaction effects in food-related neural activity were accompanied by a similar interaction in the resting functional connectivity between the anterior STS and gustatory regions of the dorsal mid-insula. These findings indicate that taste reactivity in ASD is associated with the aberrant function and functional connectivity of primary gustatory cortex and other brain regions associated with higher-order social functions.

The prominent involvement of the anterior STS, a region associated with functional deficits in other areas of ASD symptomatology (Boddaert et al., 2004; Verhoeven et al., 2010; Zilbovicius et al., 2013), suggests that multiple seemingly distinct symptoms of ASD may share a common neural etiology. Interventions, such as non-invasive brain stimulation or neurofeedback training, specifically targeting the anterior STS (Ramot et al., 2017) might thus prove beneficial for treating a variety of behaviors associated with ASD.

**Abbreviations**
AASP: Adult/adolescent sensory profile
ASD: autism spectrum disorder
BMI: body-mass index
DSM-V: the diagnostic and statistical manual of mental disorders IV
fMRI: functional magnetic resonance imaging
MRI: magnetic resonance imaging
ROI: region-of-interest
STS: superior temporal sulcus
TD: typically developing

## Author contributions

AM had full access to all the data in the study and takes responsibility for the integrity of the data and the accuracy of the data analysis.

*Study concept and design*: AM, WKS, and GW.

*Study execution and subject recruitment*: JI, SW, MC, LK, GW.

*Acquisition, analysis, or interpretation of data*: All authors.

*Drafting of the manuscript*: JAA, AM.

*Critical revision of the manuscript for important intellectual content*: All authors.

*Statistical analysis*: JAA, CR.

*Obtained funding*: AM.

*Administrative, technical, or material support*: JI, MC, SW, CR.

*Study supervision*: AM.

## Appendix A Supplementary data

Supplemental Methods, Supplemental Results, Tables S1-S3. Image 1
