# Supplementary material for: Neural correlates of taste reactivity in autism spectrum disorder
Source: Neuroimage Clin. 2018 Apr 4;19:38–46. doi: 10.1016/j.nicl.2018.04.008 (PMC6051474; doi:10.1016/j.nicl.2018.04.008)
Supplement: Supplementary file 1 — Supplemental Methods, Supplemental Results, Tables S1-S3. [file mmc1.docx]

1. **Supplemental Methods**

*1.1 Supplemental Design Information*

*1.1.1 Taste Assessment:* A member of the research team administered 0.4mL of four solutions with a different concentration of sucrose (0.1M, 0.3M, 0.45M, 0.6M sucrose). Subjects were asked to swallow each solution and then rate it on four domains—intensity, sweetness, pleasantness, and unpleasantness—on a scale of 1 (lowest possible) to 100 (highest possible) using an iPad survey. Subjects were administered each solution a total of three times in a counterbalanced order, drinking a sip of water after rating one solution and prior to tasting the next one. The experimenter repeated this procedure with four tasteless, neutral solutions, which were varying dilutions (25%, 50%, 75% or 100%) of an artificial ‘saliva’ consisting of 25 mM (1.9 mg/mL) potassium chloride and 2.5 mM (210 mcg/mL) sodium bicarbonate. The neutral solution was used as a control stimulus, as it closely matches the properties of human saliva and mimics all oral somatosensory properties of the sweet solution, yet has no taste. Participants tasted and rated each neutral solution three times. The ordering of the taste assessment was balanced so that half of the participants tasted sweet solutions first, and half started by tasting neutral solutions.

After completion of the tastant ratings, participants completed a two-option forced-choice period, where they were asked to indicate which sweet solution they considered the most pleasant, or which neutral solution tasted the most like nothing. The experimenter chose which sweet and neutral solution to administer in the scanner based primarily on the outcome of these forced-choice periods. Since the gustatory mapping task administered a high volume of sweet and neutral solutions over several scanning runs, the research team wanted to be sure that the subject would not be overwhelmed by the buildup of taste in their mouth. Therefore, in cases where the subject rated their preferred solution (during the forced-choice period) as extremely intense and/or sweet during the prior rating period, the experimenter chose a lower molarity solution after assessing ratings on all four scales.

*1.1.2 Food Picture Task*: During this task, subjects were instructed to press a button when two consecutive pictures contained objects with the same name (for example, two consecutive cheeseburger images), which required only basic-level conceptual object naming. Both food and non-food pictures were normed for both high naming accuracy and identical typicality in a previous study (Simmons et al., 2013). All pictures were presented for 2.5s in a pseudo-random order optimized for fMRI task design using optseq2 (http://surfer.nmr.mgh.harvard.edu/optseq/). Images were followed by variable duration interstimulus intervals (ISI; 2.5 – 12.5s), during which a black fixation cross appeared against a grey background. The varied, or ‘jittered’, duration of ISIs is a common practice in fMRI experimental design (Ollinger et al., 2001) that reduces serial correlation between events and enhances the precision of the BOLD response model to these trials.

*1.1.3 Gustatory Mapping Task*: During the Gustatory Mapping task, subjects received sweet and neutral tastant solutions (described above) delivered by an MR-compatible tastant delivery system, while undergoing fMRI scanning. Prior to scanning, subjects underwent a taste assessment to determine which concentration of each solution would be used in the scanner (see Taste Assessment above).

During a tastant delivery event sequence, the subject saw a word-cue for 5s indicating the imminent delivery of one of the two sweet tastants (“sweet” or “standard”) or the tasteless solution (“neutral”). Cues always accurately predicted the type of solution to be delivered. The cue words were replaced with the word “taste” for 5s, during which time the subject received the tastant indicated by the word-cue. After a variable duration (2.5-12.5s) ISI the word “wash” appeared on the screen for 2.5s and the subject received 0.8ml of the neutral solution to wash away the preceding tastant. Immediately after the wash period, the word “swallow” appeared, at which time the subject swallowed the fluid in his/her mouth. Tastant delivery events occurred 18 times in each scanning run, 6 times for sweet, 6 times for standard sweet, and 6 times for neutral tastants.

During cue-only events, subjects saw the word-cues presented for 5s, without the subsequent delivery of fluid. Cue-only events were followed by a variable duration (2.5-12.5s) ISI. These free-standing “cue-only” events occurred 12 times in each scanning run (4 times for sweet, 4 times for standard sweet, and 4 times for neutral), and were modeled in the design matrix along with the cues in the tastant delivery events, to allow for mathematically deconvolving the response to the cues from the response to the tastants themselves (Ollinger et al., 2001). Likewise, freestanding wash/swallow events were also included to allow for the deconvolution of tastant responses from wash/swallow responses. Freestanding wash/swallow events were also followed by a variable duration ISI. The visual cues for each event and ISIs were presented in black font against a white background. Each of the three Gustatory Mapping task scans lasted 620s (10min 20s). This task has previously been employed by our lab to map gustatory responsive regions of the cerebral cortex (Avery et al., 2017; Avery et al., 2015; Simmons et al., 2013).

Tastant solutions were kept at room temperature in pressurized syringes and fluid delivery was controlled by pneumatically-driven pinch-valves that released the solutions into polyurethane tubing that ran to a plastic gustatory manifold attached to the head coil. The tip of the mouthpiece was small enough to be comfortably positioned between the subject’s teeth. This insured that the tastants were always delivered similarly into the mouth. The pinch valves that released the fluids into the manifold were open and closed by pneumatic valves located in the scan room, which were connected to a stimulus delivery computer running LabView (National Instruments, Austin, TX) software, which controlled the precise timing and quantity of tastants dispensed to the subject during the scan.

*1.2 Imaging Parameters*

fMRI data was collected at the NIMH fMRI core facility at the NIH Clinical Center using a GE HDX 3 Tesla MRI scanner (GE Healthcare, Milwaukee, Wisconsin) and a Nova 16-channel receive-only head coil (or a GE 8-channel head coil for the resting-state scan), with a sensitivity encoding factor of 2 used to minimize echoplanar image (EPI) distortions. A total of 139, 248, and 206 EPI volumes were acquired for the Food Picture, Gustatory Mapping, and resting-state tasks, respectively. Each volume consisted of 44 2.8-mm axial slices (echo time (TE) = 27 ms, repetition time (TR) = 2500 ms, flip angle = 90 degrees, voxel size = 3.4375 × 3.4375 × 2.8 mm). To minimize susceptibility artifact in ventromedial prefrontal regions, the axial (AC-PC) plane was acquired at an oblique angle (15 degrees from plumb) in scanner coordinate space. Prior to EPI scans, a high-resolution T1-weighted magnetization-prepared rapid acquisition gradient-echo (MPRAGE) sequence with SENSE was used to provide an anatomical reference for the fMRI analysis (TE = 2.7 ms, TR = 7.24 ms, flip angle = 12 degrees, voxel size = 0.937 × 0.937 × 1.2 mm).

Visual stimuli for both tasks were projected onto a screen located inside the scanner bore and viewed through a mirror system mounted on the head-coil. Stimulus presentation was controlled using E-Prime 2.0 software (Psychology Software Tools, Pittsburgh, PA). Behavioral data acquired during scanning was collected using Current Designs (Philadelphia, PA) fiber optic response boxes.

*1.3 Image Preprocessing*

*1.3.1 AFNI Preprocessing:* All fMRI pre-processing was performed in AFNI (<http://afni.nimh.nih.gov/afni)>. The FreeSurfer software package (<http://surfer.nmr.mgh.harvard.edu/>) was additionally used for anatomical surface construction and parcellation. **The specific sequence of pre-processing steps used in this pipeline allows for direct comparison with previous fMRI data collected using these task paradigms** (Avery et al., 2017; Avery et al., 2015; Simmons et al., 2016; Simmons et al., 2013). The first 4 volumes of each EPI time-course were excluded from data analysis to allow the fMRI signal to reach longitudinal equilibrium, and a slice timing correction was then applied to the remaining volumes of each EPI scan. A de-spiking interpolation algorithm (AFNI’s 3dDespike) was also used to remove transient signal spikes from the EPI data. All EPI volumes were then registered to a base EPI volume using a 6-parameter (3 translations, 3 rotations) motion correction algorithm, and the motion estimates were saved for use as regressors in the subsequent statistical analyses. Volume registration and spatial normalization to Talairach space were implemented in the same transformation step, in order to minimize the number of interpolation steps performed on EPI data. Following this, smoothing with a 6mm full width at half maximum Gaussian kernel was performed, and the signal intensity for each EPI volume was normalized to reflect percent signal change from each voxel’s mean intensity across the time-course.

The data collected during both the Food Picture and the Gustatory Mapping task were separately analyzed at the single-subject level using multiple linear regression models in AFNI’s 3dDeconvolve. For the Gustatory Mapping task, the regression model included 5 regressors for 1) the combined sweet and standard sweet word-cues, 2) neutral word-cue, 3) combined sweet and standard sweet tastants, 4) neutral tastant, and 5) wash/swallow events. These regressors were constructed by convolution of a gamma-variate hemodynamic response function with a boxcar function having a 5-second width beginning at the onset of each trial period. The regressors for food and non-food images in the Food Picture task were modeled separately using a gamma variate function beginning at the onset of the picture stimulus from each image category, food or non-food object. The regression model for both tasks also included regressors of non-interest to account for each run’s mean, linear, quadratic, and cubic signal trends, as well as the 6 normalized motion parameters (3 translations, 3 rotations) computed during the volume registration preprocessing.

*1.3.2 Resting State Preprocessing:* Preprocessing of the resting-state scans was performed using a modified version of the ANATICOR method (Jo et al., 2010). Using the high-resolution anatomical scan, masks of the subject’s ventricles and white matter were constructed using FreeSurfer (http://surfer.nmr.mgh.harvard.edu/). After resampling the masks to the same resolution as the EPI, each mask was then eroded by a single voxel in each direction to prevent partial volume effects that might include signal from gray matter voxels in the mask. The average time course during the resting-state scan was extracted from the ventricle mask and subsequently used to account for any components of the MR signal due to cerebrospinal fluid in the ventricles. Next, local physiological noise present in white matter was estimated using the AFNI program 3dLocalstat, which calculated the average signal time-course for all white matter voxels within a 1.5 cm radius of each gray matter voxel. **Respiration and cardiac data were collected by pulse-oximetry and respiration belt recording during scanning. These sources of physiological noise were directly measured and regressed from the resting-state data using RETROICOR (Glover et al., 2000) and respiration volume per time (RVT)(Birn et al., 2008), as the original frequencies were aliased into the data range at a TR of 2.5 and would not be appropriately removed by band-passing techniques.** The first three principal components of the white matter and ventricle signal were also extracted and used as regressors (aCompCorr) (Muschelli et al., 2014). Additional regressors of non-interest were constructed from the mean, linear, quadratic, and cubic signal trends, as well as the 6 normalized motion parameters (3 translations, 3 rotations) computed during the image registration preprocessing. The time-course for these nuisance variables were subtracted from each resting-state voxel time-course using the AFNI program 3dTproject, yielding a residual time-course for each voxel. Following this step, the residual EPI time series was then spatially transformed to Talairach space, smoothed, and then scaled to percent signal change as above.

*1.4 Motion Censoring*

In addition to volume registration, motion-censoring algorithms were also implemented to guard against potential artifactual confounds induced by uncontrolled subject motion(Power et al., 2012). Briefly, the Euclidean-normalized derivative of the subject’s motion parameters was calculated for each TR, and a list of time points was created in which that value was greater than 0.3 (roughly 0.3mm motion; 0.2 for resting state scans). The TRs within this list were then censored during the subject-level regression analysis. Additionally, any subject with an average Euclidean-normalized derivative of greater than 0.2 during any of the tasks (0.3 for the Gustatory Mapping task to account for increased head motion due to wash/swallow periods) was excluded from the group-level analysis. Five subjects were thus excluded from Gustatory Mapping task analysis (4 ASD & 1 TD subjects; leaving 17 ASD and 20 TD subjects), 1 ASD subject was excluded from the Food Picture task analysis, and 1 ASD subject was excluded from the resting-state analysis, due to excessive head motion.

*1.5 Behavioral Analyses*

Demographic and behavioral measures of ASD and TD participants (Age, IQ, BMI, and AASP scores) were compared between groups using two-sample t-tests. Data collected from participants’ ASA-24 assessments were used to calculate the total kilocalorie consumption prior to scanning, as well as the Healthy Eating Index (HEI) (Guenther et al., 2013), an overall measure of dietary health. These measures were also compared between groups, to account for potential differences in diet or pre-scan caloric consumption. The pre-scan taste assessment was scored (similarly to (Damiano et al., 2014)) by calculating the average of log-transformed participant ratings of the intensity, sweetness, pleasantness, and unpleasantness of sweet solutions, and then identifying the slope of those ratings with increasing sucrose concentration. Those slopes were then compared between groups using a two-sample t-test.

To identify how the taste reactivity scale, derived from the taste-specific AASP questions, related to the more commonly measured AASP quadrant scores, we calculated Pearson correlations between taste reactivity scores and those quadrant scores (sensory sensitivity, sensory avoiding, low registration, sensation seeking) after first subtracting out any of the taste-specific items from those quadrant scores. Additionally, in order to assess the relationships between taste reactivity and both the quality of subjects’ diets as well as perceptual ratings of the sweet tastants, separate Pearson correlations were calculated between the derived taste scale of the AASP and the ASA-24 Healthy Eating Index, and the slopes of taste intensity, sweetness, pleasantness, and unpleasantness measured during the taste test, respectively.

*1.6 Imaging Analyses*

*1.6.1 Gustatory Mapping task:* First, we created a mask of the left and right lobes of the insular cortex, using anatomical masks generated by FreeSurfer parcellations of the AFNI N27 atlas brain in Talairach space. We applied this bilateral insula mask to the contrast ‘All tastants (both sweet + neutral) vs. 0’, using the combined Gustatory Mapping task fMRI data from all participants, to identify insula regions responsive to stimulation by all tastants (both sweet and neutral). In order to clearly define the distinct anterior and mid-insula regions responsive to tastant stimulation, we thresholded the activation within this contrast at a p-value of p < 5x10^-8^ (FDR corrected p-value << 0.001). Within each of these four insula ROIs (see Figure 1) we extracted the beta coefficients for the sweet vs. neutral tastant contrast using the AFNI program 3dLocalstat and then compared those values between groups using a group x region ANOVA, within the R statistical software package ([www.r-project.org)](http://www.r-project.org)).

In the second approach, we performed a whole-brain voxel-wise analysis, using data from the Gustatory Mapping task, to compare brain activation for sweet vs. neutral tastants between ASD and TD subjects. The individual subject regression coefficients for the contrast sweet vs. neutral tastant were compared at the whole-brain level in a two-sample t-test using the AFNI program 3dttest++.

We also examined whether behavioral measures of taste reactivity would underlie any differences between ASD and neurotypical subjects, at the ROI and whole brain level. Within the insula ROIs, we performed an analysis of covariance (ANCOVA) to identify the interaction of group and taste reactivity, as measured by the taste-specific items from the Adolescent/Adult Sensory Profile (AASP).

At the whole-brain level we used the AFNI program 3dLME to identify any regions in the brain exhibiting a significant relationship between taste reactivity and the response to sweet vs. neutral tastants. We tested these relationships separately within both ASD and TD groups. We also tested the group x taste reactivity interaction, which identified brain regions where the slope of the relationship between taste reactivity and tastant response differed between groups. We corrected the resulting whole-brain statistical maps for multiple comparisons using a cluster-size FWE correction as described below.

*1.6.2 Food Pictures task:* We next examined whether brain regions that exhibited a significant interaction to taste stimuli during the Gustatory Mapping task would also exhibit a similar interaction within the Food Picture task. Using the clusters exhibiting significant group x taste reactivity interaction effects during the Gustatory Mapping task (see above; Figure 2) as ROIs, we extracted the beta coefficients for the task conditions from the Food Picture task and performed an analysis of covariance, as above, to identify the interaction of group and taste reactivity on the response to food vs. object pictures.

*1.6.3 Resting-state functional connectivity analyses:* The ROIs exhibiting significant group x taste reactivity interaction effects during the Gustatory Mapping task (Figure 2) were used as seeds for a resting-state functional connectivity analysis, to further explore the interaction of diagnostic criteria (i.e. group differences) and taste reactivity in ASD and TD subjects. The AFNI program 3dmaskave was used to extract the average time-course of the pre-processed resting-state data within each ROI seed. Next, maps of the correlation coefficient between the seed time-course and all other voxels in the brain were produced using a voxel-wise correlation analysis. The r-value maps made by this process were then Fisher-transformed into z-score maps for use in the group analysis.

At the group level, we used the AFNI program 3dttest++ to identify the interaction of group (ASD vs. TD) and taste reactivity on the functional connectivity between each of our seed regions and the rest of the brain. This allowed us to identify whether the intrinsic functional connectivity of our seed regions exhibited an interaction effect similar to that seen in the Gustatory Mapping task data. Resultant statistical maps were cluster-size corrected for multiple comparisons at p < 0.05 as described above. The statistical maps separately generated for the left and right STS regions were observed to be qualitatively and quantitatively similar (voxel-wise eta^2^ similarity metric = 0.9), so the seed time-courses for the bilateral STS regions were averaged for the subject-level correlation analysis, from which the final bilateral STS statistical map was generated.

*1.7 Multiple Comparison Correction*

Contrast maps were cluster-size corrected for multiple comparisons as follows. An initial voxel-wise p-value threshold of p < 0.001 was applied to the statistical map, and a cluster-size correction of p < 0.05 was implemented using AFNI’s 3dClustsim. All contrast maps were corrected within a mask in which the group average temporal signal-to-noise ratio of the EPI data was at least 40, to ensure that the fMRI signal within these brain regions allowed for the reliable detection of task effects (Murphy et al., 2007). Importantly, recent evidence has suggested that Gaussian smoothness estimates drastically underestimate the actual spatial auto-correlations present within fMRI data, leading to underestimations of the minimum cluster-size required to achieve family-wise error (FWE) correction(Eklund et al., 2016). In order to more precisely estimate the cluster-sizes required to achieve FWE correction, revised versions of AFNI’s 3dFWHMx and 3dClustsim were used to generate smoothness and cluster-size estimates using a spherical non-Gaussian spatial autocorrelation function. Using these smoothing parameters and this initial p-value threshold, this method has been demonstrated to produce corrected cluster size values approximately equal to those achieved through non-parametric permutation methods (Cox et al., 2017).

1. **Supplemental Results**

*2.1 Additional Behavioral Results*

Groups did not differ in the average slopes of their log-transformed intensity, sweetness, pleasantness, and unpleasantness ratings (all p-values > 0.44). Intensity and sweetness slopes for both groups were positive and greater than zero, indicating that participants perceived increasing concentrations of sucrose as both sweeter and more intense. We observed a modest but significant relationship between taste reactivity and intensity slopes (r(40) = 0.31; p < 0.03), such that individuals with greater self-reported taste reactivity rated sweet tastants as much more intense with increasing sucrose concentration. We did not observe any similar relationships between taste reactivity and sweetness, pleasantness, or unpleasantness ratings (p > 0.20). Taste reactivity scores were positively related to the overall AASP quadrant scores of Sensory Sensitivity (r(40) = 0.61; p < 0.001), Sensory Avoiding (r(40) = 0.41; p < 0.003), and Low Registration (r(40) = 0.45; p < 0.001), but not Sensation Seeking (r(40) = -0.13; p = 0.42).

*2.2 BMI and IQ effects*

As BMI and IQ differed significantly between groups (Table 1), we performed a series of analyses to examine whether IQ and BMI had a significant effect upon our neuroimaging results. These analyses of variance, which compared the linear models computed with and without the inclusion of IQ and BMI as covariates, found that adding these variables did not significantly improve the amount of variance explained either in tastant responses in the insular cortex (p = 0.47) or in the response to food vs. object pictures in the clusters from the voxel-wise interaction map (Figure 2, Table 3; p = 0.61). Likewise, the voxel-wise statistical maps generated through analyses of the gustatory mapping or resting-state data were not qualitatively affected by the addition of BMI or IQ as covariates.

**As an additional supplemental analysis, we examined the contribution of varying tastant concentrations within subjects, during the preferred ‘sweet’ tastant trials, to the variance explained by group analyses of gustatory activity within the brain. We observed that the inclusion of sucrose molarity as a group-level covariate (delivered during ‘sweet’ trials) did not significantly affect the variance explained by analyses of the tastant response within the insular cortex (p=0.37) or within the clusters of the interaction mask (p=0.33).**

*2.3 Additional insula ROI analyses*

In order to confirm that the pattern of effects observed in the Gustatory Mapping task data within our insula regions was not due to any bias in ROI selection, we performed an additional set of analyses within an independent set of ROIs in the anterior and mid-insula, derived from a meta-analysis of gustatory human neuroimaging studies (Veldhuizen et al., 2011). As before, we observed no effects of group within the insula regions (p=0.90), but we did observe positive relationships between taste reactivity and the response to sweet vs. neutral tastants in both regions of the left insula (anterior, r(15)=0.48; p=0.05; mid-dorsal, r(15)=0.56; p<0.02) for the ASD, but not TD subjects (p > 0.25).

**References**

Avery, J.A., Gotts, S.J., Kerr, K.L., Burrows, K., Ingeholm, J.E., Bodurka, J., Martin, A., Kyle Simmons, W., 2017. Convergent gustatory and viscerosensory processing in the human dorsal mid-insula. Hum Brain Mapp 38, 2150-2164.

Avery, J.A., Kerr, K.L., Ingeholm, J.E., Burrows, K., Bodurka, J., Simmons, W.K., 2015. A common gustatory and interoceptive representation in the human mid-insula. Hum Brain Mapp 36, 2996-3006.

Birn, R.M., Murphy, K., Bandettini, P.A., 2008. The effect of respiration variations on independent component analysis results of resting state functional connectivity. Hum Brain Mapp 29, 740-750.

Cox, R.W., Chen, G., Glen, D.R., Reynolds, R.C., Taylor, P.A., 2017. fMRI clustering and false-positive rates. Proc Natl Acad Sci U S A 114, E3370-E3371.

Damiano, C.R., Aloi, J., Burrus, C., Garbutt, J.C., Kampov-Polevoy, A.B., Dichter, G.S., 2014. Intact hedonic responses to sweet tastes in autism spectrum disorder. Research in Autism Spectrum Disorders, pp. 230-236.

Eklund, A., Nichols, T.E., Knutsson, H., 2016. Cluster failure: Why fMRI inferences for spatial extent have inflated false-positive rates. Proc Natl Acad Sci U S A 113, 7900-7905.

Glover, G.H., Li, T.Q., Ress, D., 2000. Image-based method for retrospective correction of physiological motion effects in fMRI: RETROICOR. Magn Reson Med 44, 162-167.

Guenther, P.M., Casavale, K.O., Reedy, J., Kirkpatrick, S.I., Hiza, H.A., Kuczynski, K.J., Kahle, L.L., Krebs-Smith, S.M., 2013. Update of the Healthy Eating Index: HEI-2010. J Acad Nutr Diet 113, 569-580.

Jo, H.J., Saad, Z.S., Simmons, W.K., Milbury, L.A., Cox, R.W., 2010. Mapping sources of correlation in resting state FMRI, with artifact detection and removal. Neuroimage 52, 571-582.

Murphy, K., Bodurka, J., Bandettini, P.A., 2007. How long to scan? The relationship between fMRI temporal signal to noise ratio and necessary scan duration. Neuroimage 34, 565-574.

Muschelli, J., Nebel, M.B., Caffo, B.S., Barber, A.D., Pekar, J.J., Mostofsky, S.H., 2014. Reduction of motion-related artifacts in resting state fMRI using aCompCor. Neuroimage 96, 22-35.

Ollinger, J.M., Shulman, G.L., Corbetta, M., 2001. Separating processes within a trial in event-related functional MRI I. The Method. Neuroimage 13, 210-217.

Power, J.D., Barnes, K.A., Snyder, A.Z., Schlaggar, B.L., Petersen, S.E., 2012. Spurious but systematic correlations in functional connectivity MRI networks arise from subject motion. Neuroimage 59, 2142-2154.

Simmons, W.K., Burrows, K., Avery, J.A., Kerr, K.L., Bodurka, J., Savage, C.R., Drevets, W.C., 2016. Depression-Related Increases and Decreases in Appetite: Dissociable Patterns of Aberrant Activity in Reward and Interoceptive Neurocircuitry. Am J Psychiatry, appiajp201515020162.

Simmons, W.K., Rapuano, K.M., Kallman, S.J., Ingeholm, J.E., Miller, B., Gotts, S.J., Avery, J.A., Hall, K.D., Martin, A., 2013. Category-specific integration of homeostatic signals in caudal but not rostral human insula. Nat Neurosci 16, 1551-1552.

Veldhuizen, M.G., Albrecht, J., Zelano, C., Boesveldt, S., Breslin, P., Lundstrom, J.N., 2011. Identification of human gustatory cortex by activation likelihood estimation. Hum Brain Mapp 32, 2256-2266.

**Table S1. Gustatory-responsive regions of the insular cortex**

| Location | Peak Coordinates | | | Peak t | Cluster p-value | Volume (mm^3^) |
| --- | --- | --- | --- | --- | --- | --- |
|  | X | Y | Z |  |  |  |
| R Anterior Insula | +35 | -5 | +10 | 8.55 | < 0.01 | 1,112 |
| L Anterior Insula | -31 | +15 | +10 | 10.72 | < 0.01 | 1,024 |
| L Mid Insula | -33 | -3 | +12 | 8.84 | < 0.01 | 944 |
| R Mid Insula | +35 | -5 | +10 | 8.74 | < 0.01 | 400 |

**Table S2. Group by taste reactivity interactions in the response to food vs. object pictures**

|  | **Group x Taste Reactivity** | |
| --- | --- | --- |
| **Region** | F | p |
| L anterior superior temporal sulcus | 8.80 | < 0.006 |
| R anterior superior temporal sulcus | 4.97 | < 0.05 |
| R medial striatum | 9.95 | < 0.005 |
| L fusiform gyrus | 6.95 | < 0.05 |
| Dorsomedial anterior cingulate / prefrontal cortex | 4.27 | < 0.05 |

**Table S3. Brain regions exhibiting an interaction between group and taste reactivity in the resting-state connectivity to the anterior superior temporal sulcus**

| Location | Peak Coordinates | | | Peak t | Cluster p-value | Volume (mm^3^) |
| --- | --- | --- | --- | --- | --- | --- |
|  | X | Y | Z |  |  |  |
| R post-central gyrus | +55 | -27 | +28 | 4.74 | < 0.01 | 1,976 |
| L dorsal mid-insular cortex | -39 | -3 | +6 | 4.99 | < 0.01 | 1,656 |
| R dorsal mid-insular cortex | +39 | -9 | +8 | 5.14 | < 0.01 | 1,504 |
| L inferior parietal cortex | -35 | -53 | +50 | 5.44 | < 0.01 | 1,352 |
